# Effectiveness of Lateral Elbow Tendinopathy Treatment Depends on the Content of Biologically Active Compounds in Autologous Platelet-Rich Plasma

**DOI:** 10.3390/jcm11133687

**Published:** 2022-06-27

**Authors:** Maciej Dejnek, Helena Moreira, Sylwia Płaczkowska, Ewa Barg, Paweł Reichert, Aleksandra Królikowska

**Affiliations:** 1 Clinical Department of Trauma and Hand Surgery, Department of Trauma Surgery, Faculty of Medicine, Wroclaw Medical University, 50-556 Wroclaw, Poland; pawel.reichert@umw.edu.pl; 2 Department of Medical Science Foundation, Faculty of Pharmacy, Wroclaw Medical University, 50-556 Wroclaw, Poland; helena.moreira@umw.edu.pl (H.M.); ewa.barg@umw.edu.pl (E.B.); 3 Teaching and Research Diagnostic Laboratory, Department of Laboratory Diagnostics, Faculty of Pharmacy, Wroclaw Medical University, 50-556 Wroclaw, Poland; sylwia.placzkowska@umw.edu.pl; 4 Ergonomics and Biomedical Monitoring Laboratory, Department of Physiotherapy, Faculty of Health Sciences, Wroclaw Medical University, 50-355 Wroclaw, Poland; aleksandra.krolikowska@umw.edu.pl

**Keywords:** platelet-rich plasma (PRP), tennis elbow, lateral elbow enthesopathy, lateral epicondylitis, growth factors, cytokines

## Abstract

Autologous platelet-rich plasma (PRP) injection is an alternative treatment option for patients with lateral elbow tendinopathy. The treatment is supposed to accelerate tissue regeneration by providing high concentrations of growth factors derived from platelets. The aim of the study was to assess the relationship between the content of biologically active compounds in PRP and the clinical effect of the treatment. Thirty patients with lateral elbow tendinopathy treated with a single PRP injection, were evaluated. The pain intensity (measured by a visual analogue scale (VAS)), the pressure pain threshold (PPT), the grip strength and strength of the main arm and forearm muscle groups, and the functional outcome (measured by the Disability of Arm, Shoulder and Hand (DASH) and Patient-Rated Tennis Elbow Evaluation (PRTEE) questionnaires), were assessed before PRP injection and at one- and three-months follow-up. Flow cytometry measurements of the growth factors and inflammatory cytokines in PRP were performed, and the results were used to establish the relationship between those molecules and the clinical outcome. After three months from the intervention, the minimal clinically important difference in pain reduction and functional improvement was observed in 67% and 83% of patients, respectively. Positive correlations were found between the extent of pain reduction after three months and concentrations in the PRP of platelets, epidermal growth factor (EGF), vascular endothelial growth factor, and platelet-derived growth factors. The concentration of EGF in the PRP significantly correlated with an improvement in grip strength, strength of wrist extensors, and the size of functional improvement measured by the PRTEE. The local injection of PRP is a safe and effective treatment option for lateral elbow tendinopathy, and the clinical outcome is correlated with concentrations of its biologically active compounds.

## 1. Introduction

Lateral elbow tendinopathy is a degenerative condition of proximal attachment of extensor muscles to the humeral epicondyle [1]. Among the best-described causes of the disease are repeated movements of the upper limb and lifting heavy objects [2]. Although the most commonly used name is ‘tennis elbow’, the disease is not always connected to sports activity [3]. It mainly occurs in manual workers, who perform multiple flexion and extension as well as torsional wrist movements and forceful griping at work [4]. The disease affects 1–3% of the population, of both sexes, most often in the age between 40 and 60 years [5].

From the pathophysiological point of view, as a result of repeated microtraumas, abnormal angiofibroblastic remodeling develops on the site of muscle attachment, leading to increased pain sensation [6]. These changes result from incorrect repairing processes, consequentially interrupted by overload injuries [7] and extensor carpi radialis brevis (ECRB) and common extensor (CE) origins are mainly involved [6]. Conservative treatment is the method of choice at the beginning of the disease and avoiding limb overloading, changing harmful movement patterns at work, cooling, stretching and medications for pain control could be sufficient for the early stages [8]. A self-limiting character of the disease leads to spontaneous remission within a few weeks, and in most of the patients, the pain will resolve finally within a year [4]. Unfortunately, in some patients, severe pain intensity makes it impossible to perform simple everyday activities.

For patients in whom the first line treatment has failed, no sufficiently reliable treatment has been developed so far. The most commonly used second line treatment method is the local injection of corticosteroids [9]. Unfortunately, such treatment, although very effective in the short term, is associated with the highest rate of recurrence and numerous complications [10].

The treatment targeted at enhancing healing is the injection of autologous platelet-rich plasma (PRP) [11]. The procedure involves delivering to the site of injury, previously prepared patient’s plasma with a higher than baseline platelet content. These platelets are supposed to locally release a large number of cytokines and growth factors, which should stimulate the natural repair processes [11]. Among the cytokines released from the platelets’ α-granules, the best described are platelet-derived growth factors (PDGF), vascular endothelial growth factor (VEGF), epidermal growth factor (EGF), fibroblast growth factor (FGF), and transforming growth factor-β1 [12]. Many other cytokines are also involved in healing processes and among the most important are Interferon- γ and -α (IFN-α, and IFN-γ), numerous interleukins (IL), metalloproteinases, and chemoattractant proteins [12]. Moreover, the influence of individual cytokines on the healing processes has been studied; however, evaluating their effects on living organisms is challenging due to their numerous mutual interactions, both stimulating and inhibiting.

There are many doubts about the efficacy of PRP injection in lateral elbow tendinopathy. Most publications have presented significant improvements, being better than corticosteroid injection at more than three months after treatment or even being equal to surgery [13,14,15,16]. On the other hand, some studies have reported similarly good outcomes after saline or autologous blood injection [14,17] while three months appeared to be the cut-off point for positive PRP treatment results. Conversely, patients, who are still suffering after an extended period, often do not want to wait any longer and start looking elsewhere for help [18]. An additional problem in assessing the effectiveness of treatment is the great variety of methods for obtaining PRP. Many commercial PRP kits differ in the way they separate the platelets from other cellular components, the amount of blood to be drawn from the patient, and the platelets’ activation method [19]. This leads to a PRP with a different density of platelets or white blood cells and, consequently, a different density of growth factors and cytokines [20]. Previous studies have shown that both platelets and leukocytes in PRP can correlate with higher levels of growth factors; therefore, we decided to use a commercial kit that allows for obtaining both a high platelet and leukocyte content. Additional potential benefits of the use of a leukocyte-rich PRP include its antimicrobial properties and the ability to influence an immune response. The existing concerns about the negative impact on tissue healing due to an ability to stimulate catabolic reactions, and the release of pro-inflammatory cytokines and proteinases have not been confirmed in in vivo studies [15,16].

The aim of the study was to determine the relationship between the content of selected growth factors or inflammatory cytokines in PRP samples and the effectiveness of the PRP injection treatment in patients with lateral elbow tendinopathy. We hypothesized that the greater the content of growth factors in the injected PRP, the greater the pain reduction, functional improvement, and improvement in muscle strength during three months of follow-up.

## 2. Materials and Methods

### 2.1. Ethical Standards

The study was carried out according to the Declaration of Helsinki, and was approved by the Bioethics Committee of Wroclaw Medical University (KB—26/2019, 21 January 2019). All patients agreed to participate in the study and signed an informed consent. The patients were assessed from February 2021 to May 2022 at the Department of Trauma Surgery.

### 2.2. Study Design

The study was designed as a part of a single-center, double-blinded, prospective randomized controlled trial titled ‘Evaluation of Clinical Efficacy and Comparison of Autologous Platelet Rich Plasma, Hyaluronic Acid, Corticosteroid and Saline Injections for Treating Lateral Humeral Epicondylopathy’ registered in clinicaltrials.gov under the identifier, NCT04521387. The study was double-blinded, so that the patient and the assessor evaluating the outcomes had no information about the type of treatment. The patients were not informed of the kind of injection they received, and the syringe was covered with opaque tape to mask its contents during administration. Each patient had a blood sample taken for complete blood counts, so that they could not easily guess if they were assigned to the PRP group, which was the only group that required blood donation. The only person who knew the allocation of patients to the groups was the primary supervisor, who decided to unblind the results of the three-months follow-up of patients in the PRP group to present the correlation results between the growth factors and cytokines in the current publication. The results of all group comparisons will be presented at the end of the one-year observation period in accordance with the original protocol, which is expected to be in the year 2023.

### 2.3. Population

**Figure 1:**
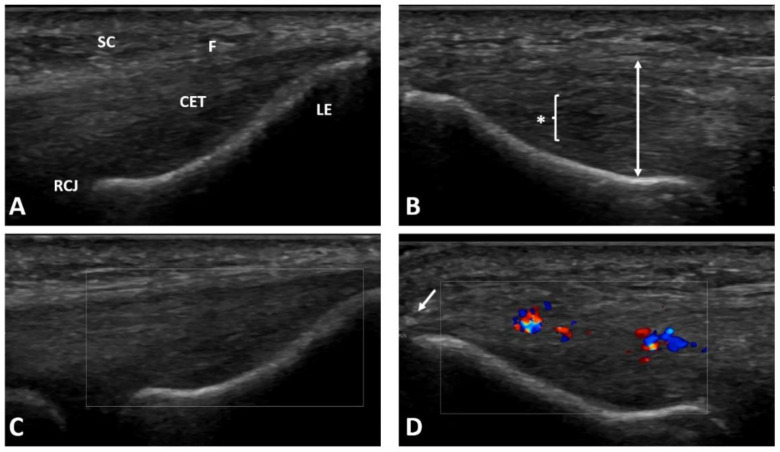
Ultrasound examination of the patient’s both elbows for lateral elbow tendinopathy confirmation: (**A**) healthy lateral epicondyle region of the contralateral elbow; (**B**) pathological common extensor tendon thickening and region with diffuse hypoechogenicity typical for lateral elbow tendinopathy; (**C**) healthy common extensor tendon without activity in color Doppler; (**D**) neoangiogenesis shown as increased activity in color Doppler in the tendinopathic region. SC—subcutaneous tissue; F—fascia; CET—common extensor tendon; LE—lateral epicondyle; RCJ—radiocapitellar joint; * and bracket—diffuse hypoechogenic region; two-head arrow—tendon thickness measurement; arrow—enthesophyte.

We enrolled patients with pain on the lateral side of the elbow joint after the failure of first line treatment for at least three months, with confirmation of lateral elbow enthesopathy in at least one provocative test (the Cozen’s test, Thomson’s test, Maudsley’s test, or Chair test) [1]. Patients with previous invasive treatment on the lateral aspect of the involved elbow (including previous injections), with hematological diseases, diabetes, gout, malignant tumors, advanced osteoarthritis of the elbow joint, nervous system diseases related to the upper limb, suspicion of an infectious process, who were pregnant, or taking medications that may affected platelet function or the coagulation system were excluded from the study. The patients learned about the possibility of taking part in the study from the University’s website and social media and during a visit at an outpatient clinic. If they volunteered to participate in the study, they were recruited after meeting the inclusion criteria at the initial visit. A bilateral X-ray of the elbow and ultrasound examination were performed to exclude other pathologies and to confirm changes typical for lateral elbow enthesopathy (see Figure 1) [21]. Information about the patients’ age, height, and weight (for body mass index, BMI calculation), type of work, duration of symptoms, smoking status, sports activity, medications, any other diseases, and previous treatments were collected during a medical interview.

### 2.4. Intervention

Under aseptic conditions, from each patient, 27 mL of blood was drawn from a cubital vein of the opposite elbow into a 30 mL syringe filled with 3 mL of anticoagulant citrate dextrose solution A (ACD-A). An additional two ml was drawn to the probe with ethylenediaminetetraacetic acid (EDTA) and was used for the complete blood count analysis using the Mindray BC-5150 automatic laboratory analyzer (Shenzhen Mindray Bio-Medical Electronics Co., Shenzhen, China).

**Figure 2:**
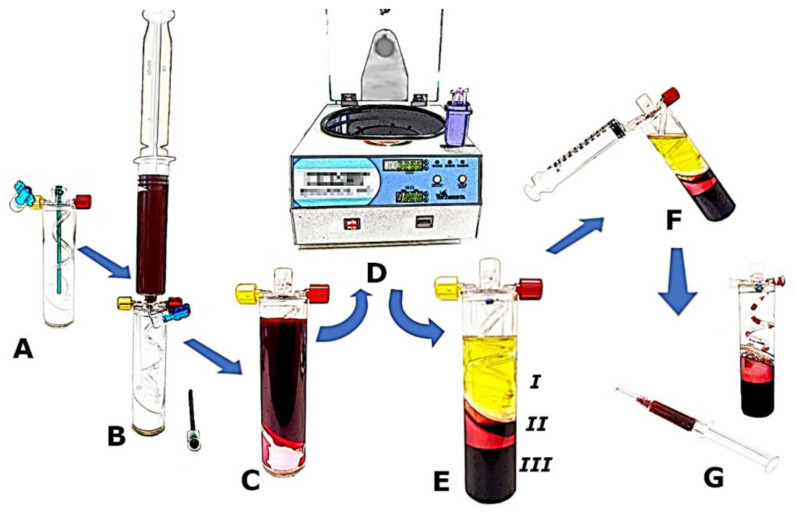
Step-by-step preparation process for obtaining LR-PRP by the Mini GPS III Platelet Concentration System (Biomet Inc., Warsaw, IN, USA): (**A**) empty separation tube; (**B**) addition of 30 mL whole blood mixed with anticoagulant; (**C**) separation tube filled with patient’s blood; (**D**) single centrifugation for 15 min; (**E**) the Mini GPS III tube after centrifugation with blood separated into three layers: *I*—platelet-poor plasma, *II*—platelet-rich plasma and buffy coat, *III*—red blood cells; (**F**) platelet-poor plasma removal; (**G**) platelet-rich plasma taken out of the tube into a syringe.

A 30 mL measure of blood mixed with anticoagulant was transferred to a Mini GPS III Platelet Concentration System (Biomet Inc., Warsaw, IN, USA) and then centrifugated for 15 min with 3200 revolutions per minute (RPM), at a force of 1740× *g*. According to the manufacturer’s protocol, about 3 mL of liquid-form leukocyte-rich platelet-rich plasma (LR-PRP) was obtained. Platelet-poor plasma was removed from the separation tube, and the RBC layer was left in the tube under the separation membrane. For better visualization, the step-by-step process of obtaining PRP by the Mini GPS III is illustrated in Figure 2. One ml of PRP was reinjected into an Eppendorf polypropylene tube for further cell content analysis, and then the samples were activated by a double freeze–thaw process for 30 min in each step according to the method proposed by Zimmermann et al. [22]. This is a relatively simple and convenient method of platelet activation, especially in the planned storage of a frozen preparation. It allows the release of growth factors in a similar way to other activation methods, including in vivo activation by contact with native collagen. In the next step, the samples were frozen and stored at −80 °C until the time of the final growth factors and inflammatory cytokines analysis. The time between the blood draw, PRP separation, injection, complete blood count analysis, activation, and freezing for further storage did not exceed 1 h. The whole process was conducted in daylight at room temperature.

**Figure 3:**
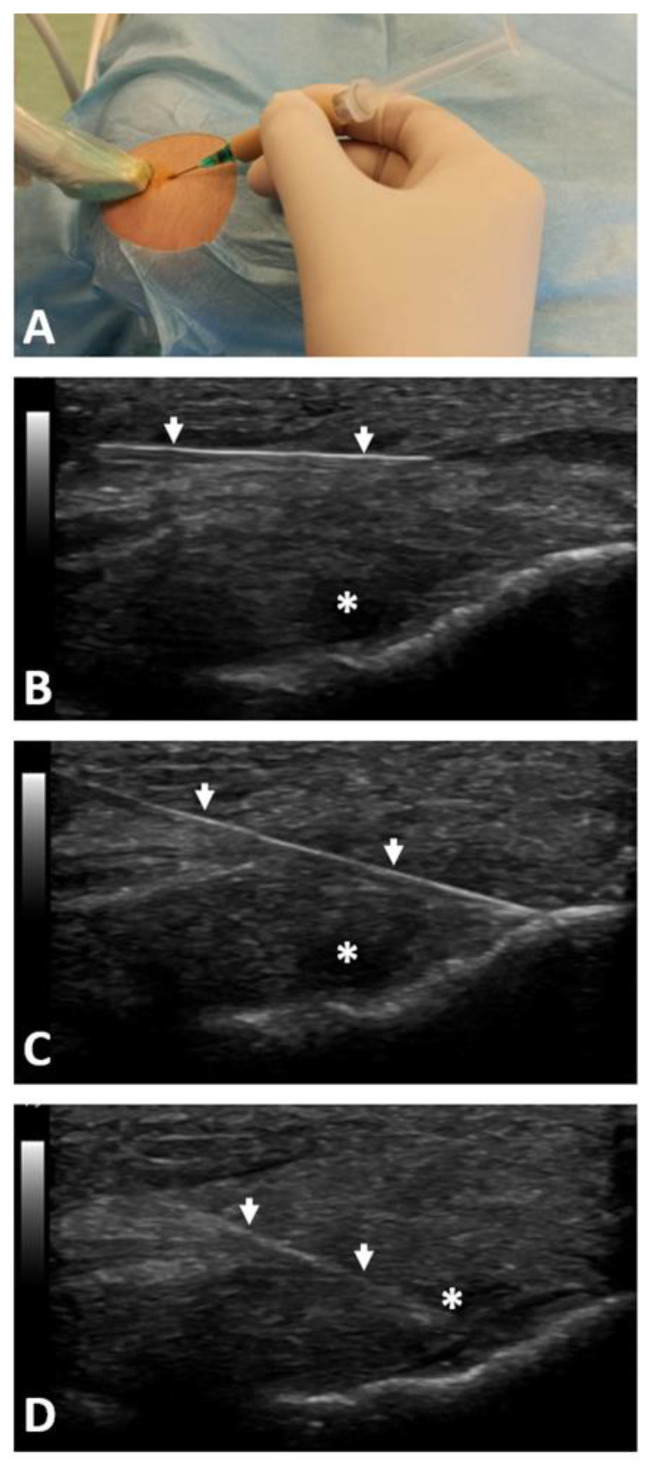
Presentation of ultrasound-guided PRP injection technique into common extensor tendon: (**A**) point of skin puncture with a needle guided by ultrasound probe in sterile conditions; (**B**) injection of 1–2 mL of 1% lidocaine in the subcutaneous region; (**C**) the peppering technique with multiple needle punctures through the fascia to the periosteum; (**D**) PRP injection into the hypoechogenic region of ECRB. PRP—platelet-rich plasma; ECRB—extensor carpi radialis brevis; arrows—view of the needle position under ultrasound; *—hypoechogenic region of the tendon.

Five minutes before the injection of PRP, 1–2 mL of local anesthetic (1% lidocaine) was injected subcutaneously into the epicondyle region. Then, under aseptic conditions, under the guidance of ultrasonography, 2 mL of non-activated, liquid LR-PRP was injected into the ECRB and CE attachment to the lateral humeral epicondyle using the peppering technique from one access through the skin and with multiple punctures (approximately 10 times) through the fascia to the periosteum. The PRP was injected above and under the fascia and into the tendon lesions identified with the ultrasonography. The 21G needle was used for that purpose. A sterile dressing was applied (see Figure 3). After injection, the patients were observed for approximately 30 min. Patients were instructed to avoid overloading activities and to start stretching exercises after a week from the injection. The inclusion of eccentric exercises to strengthen the extensors of the forearm was recommended in the case of a pain reduction below three on the visual analogue scale (VAS), not earlier than two weeks after the intervention.

### 2.5. Evaluation of Biologically Active Compounds

The two LEGENDplex multiplex immunoassays based on fluorescence-encoded beads and flow cytometric measurements were used to assess the content of cytokines and growth factors in the PRP. The first, LEGENDplex TM Custom Human 7-plex Panel (BioLegend, San Diego, CA, USA), is a custom-made panel dedicated to our study to estimate the concentration of the most important growth factors derived by platelets: transforming growth factor-β1 (TGF-β1, free active), epidermal growth factor (EGF), fibroblast growth factor-basic (FGF-basic), vascular endothelial growth factor (VEGF), hepatocyte growth factor (HGF), platelet-derived growth factor-AA (PDGF-AA), and platelet-derived growth factor-BB (PDGF-BB). The second, LEGENDplex TM Human Inflammation Panel 1 (BioLegend, San Diego, CA, USA), is included in the manufacturer’s standard offer and was used to evaluate the inflammatory cytokine content in the samples tested: Interleukin-1β (IL-1β), Interferon-α2 (IFN-α2), Interferon-γ (IFN-γ), Tumor Necrosis Factor α (TNF-α), Monocyte Chemoattractant Protein-1 (MCP-1), Interleukin-6 (IL-6), Interleukin-8 (IL-8), Interleukin-10 (IL-10), Interleukin-12p70 (IL-12p70), Interleukin-17A (IL-17A), Interleukin-18 (IL-18), Interleukin-23 (IL-23), and Interleukin-33 (IL-33).

At the time of further analysis, all samples were thawed to room temperature and centrifuged for 5 min at 2500 RPM (350× *g*) in a Micro Star 17 microcentrifuge (VWR International Company, Thermo Electron LED, Germany) and diluted 2× in the assay buffer. Then, the growth factors and cytokine content analysis was performed according to the manufacturer’s procedure for the LEGENDplex using the CyFlow Cube8 flow cytometer (Sysmex-Partec, Görlitz, Germany), applying a 488 nm laser with a 536/40 (BP) filter for the PE fluorochrome, and a 638 nm laser with 675/20 (BP) for the APC fluorochrome. The LEGENDplex TM Data Analysis Software version 8.0 (Vigene Tech Inc., Carlisle, MA, USA) was used for the analysis of the results. The concentration of each growth factor/cytokine was determined by means of a standard curve generated during the performance of the assay.

### 2.6. Evaluation of Treatment Efficacy

**Figure 4:**
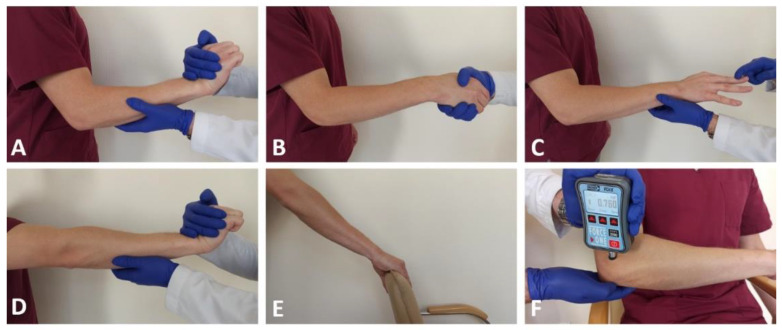
Illustration of the assessment of provocation tests and the pressure pain threshold measurement: (**A**) Cozen’s test; (**B**) Mill’s test; (**C**) Maudsley’s test; (**D**) Thomson’s test; (**E**) Chair test; (**F**) pressure pain threshold measurement.

Just before the injection and consecutively one and three months later, the patients were evaluated according to pain intensity, muscle strength, and everyday functioning. The pain intensity was assessed with the VAS, which is a visual representation of a numerical scale from 0 (no pain) to 10 (the worst pain imaginable). After a week from the injection, we asked by phone about the patients’ average pain intensity. Patients reported their average pain intensity in the VAS, of the current day and during the provocation tests. We used the five most commonly used provocation tests for tennis elbow: Cozen’s test, Mill’s test, Maudsley’s lateral epicondylitis test, Thomson’s test, and the Chair test [1]. The tests were interpreted as positive if the pain in the area of the lateral epicondyle increased during specific tasks. For the Cozen’s test, this is the extension of the wrist against resistance with the forearm pronated, and the elbow flexed to 90 degrees. In the Thomson’s test, the patient performs the same movement with the extended elbow. The starting position in the next two tests is the same as in the Cozen’s test. In the Mill’s test, the patient is trying to supinate the forearm against resistance and in the Maudsley’s test, the patient is trying to fully extend the third finger against resistance. In addition, the Maudsley’s test modification with an extended elbow was performed. The last-mentioned provocation test is the so-called Chair test, when the patient tries to lift the chair by holding the backrest with the grip, and having the elbow in extension. All these tests require contraction of the extensor muscles in the forearm, causing pain in the affected proximal attachment (see Figure 4).

The pressure pain threshold (PPT) was assessed using the Wagner FPIX 25 Pain Test Algometer (Wagner Instruments, Riverside, CT, USA). This is a digital algometer with a hard rubber tip of 1 cm^2^ surface. The algometer was applied to the site of greatest tenderness in the epicondyle area and pressed at a rate of 1 newton (N) per second. The measurement was read at the moment when the sensation of touch changed into the weakest pain sensation declared by the patient. This means that the lower the measurement reading (i.e., less pressure force needed to induce pain), the greater the tenderness. To assess the patient’s pain sensitivity, the test was also performed on the same region of the opposite limb. The test was then repeated, and the final score was calculated as the mean of two consecutive measurements for each elbow. In all cases the PTT assessment was carried out by the same rater.

Consecutively, the strength of the following muscle groups was evaluated using a microFET2 (Hoggan Scientific, Salt Lake City, UT, USA) dynamometer: elbow extensors and flexors, forearm supinators and pronators, and wrist extensors. The measurements were performed in a lying position. The tests were performed in accordance with the manufacturer’s recommendations. All the strength measurements were carried out by the same rater.

The grip strength assessment was carried out using the BIMS Digital Grip Dynamometer (Baseline, Washington, DC, USA). It was measured twice with a 1-min-long interval. The patient was comfortably placed with the elbow flexed to 90 degrees and the forearm in a neutral position resting on the armchair and asked to squeeze the dynamometer as hard as possible [23]. The patients were instructed to stop the test in the case of worsening pain symptoms, which was recorded.

The functional outcomes were measured using polish versions of two functional questionnaires: the Disability of Arm, Shoulder, and Hand Questionnaire (DASH), which is most commonly used for upper limb function evaluation, and the Patient-Rated Tennis Elbow Evaluation (PRTEE), which is best validated for the lateral elbow enthesopathy [24,25,26,27]. The DASH questionnaire consists of 30 questions about daily functioning. The patient answers each question on a 5-point scale, where 1 is the best and 5 is the worst outcome. The obtained sum is converted to a 100-point scale, taking into account the questions that the patient did not answer. The PRTEE questionnaire has two sections. The first one assessing pain intensity contains 5 questions rated from 0 (no pain) to 10 (worst imaginable pain). The second part assesses the function of the limb and includes 10 tasks rated from 0 (no difficulty) to 10 (unable to do). The final result (0–100) is the sum of the pain scale score and half of the functional scale score. In both questionnaires, the higher the score, the worse the function.

In addition, the patients were asked to assess both elbows with the Subjected Elbow Value (SEV), which was proven to be an easily administered, responsive, valid tool to assess the condition of the elbow [28]. The SEV is defined as the patient’s subjective estimation of the elbow as a percentage of a normal elbow, which would correspond to 100%.

The treatment was considered effective if a minimal clinically important difference (MCID) occurred in pain reduction, or a functional improvement between the baseline and follow-up periods. According to the literature, a MCID is equal to 1.5 points for the VAS, 15.8 points for the DASH, and 11 points for the PRTEE [16].

### 2.7. Statistical Analysis

The Shapiro–Wilk test was performed to assess the normal distribution of the results obtained. Data with a normal distribution were presented as an arithmetic mean and standard deviation (SD). Data with a non-normal distribution were described using the median and quartile distributions (Q1–Q3). The size of a treatment efficacy was presented as the change (∆) between the baseline results and those at the 1st and 3rd month (∆1 and ∆3, respectively). The significance of the differences between the subsequent follow-up periods was tested with the Student’s *t*-test for paired samples with a normal distribution and the Wilcoxon signed-rank test for nonparametric comparison of two matched samples. To assess the potential relationship between the biologically active compounds in the PRP and the efficacy of treatment, the Pearson’s correlation coefficient was established. The magnitudes of all the bivariate associations were classified as negligible (0.00–0.30), low (0.31–0.50), moderate (0.51–0.70), high (0.71–0.90), and very high (0.91–1.00). To find a significant moderate correlation (*r* ≥ 0.50) with a satisfying test power (1-β > 0.8), the required sample size was estimated at 27. For the statistical calculations, the computer software Statistica 13.3 software (TIBCO Software Inc., Pittsburgh, PA, USA) was used. The significance of the results was established at *p* < 0.05.

## 3. Results

### 3.1. Pre-Treatment Evaluation

The patients were enrolled in the study in the years 2021–2022. Their main characteristics and baseline values are presented in Table 1. All 30 patients appeared at the one and three months post-intervention follow-up visits. Among unrelated conditions, they had hypertension (*n* = 4), lumbar discopathy (*n* = 4) and depression (*n* = 2). All patients underwent ineffective first-line conservative treatment, including stretching and strengthening exercises (*n* = 21), using an orthotic device for tennis elbow (*n* = 5), manual therapy (*n* = 10), laser therapy (*n* = 4), shockwave (*n* = 2), iontophoresis (*n* = 3), and cryotherapy (*n* = 3).

The comparison between the complete blood count in whole blood and in PRP samples is presented in Table 2. The platelet concentration in PRP increased 4.38 (SD = 1.67) times than in the baseline. The concentration of WBC increased 4.60 times (SD = 1.12) than in the baseline. The RBC concentration decreased 0.21 (SD = 0.16) times than in the baseline. The mean platelet volume (MPV) in PRP was 9.67 fL (SD = 0.85). The growth factor and inflammatory cytokine concentrations in the PRP are presented in Supplementary Table S1.

### 3.2. Treatment Efficacy and Complications

No infections, neural lesions, collateral ligaments disruptions or joint cartilage damage were found during the three-month period. The main side effect was the intensification of pain, which was observed in five patients after a week, in four patients after a month, and in two patients after three months. Among these last two patients, one decided to undergo surgery, and the other decided to change their employment, in which lifting heavy objects was not required. The treatment was considered successful for pain reduction (MCID 1.5 points in the VAS) after one month in 15 patients, which increased to 20 patients after three months. After three months, pain completely disappeared only in five patients and did not change at all in two patients. A successful functional improvement measured by a difference in the DASH (MCID 15.8 points) after one and three months from injection, was established for 11 and 20 patients, respectively. A successful functional improvement measured by a difference in the PRTEE (MCID 11 points) after one and three months from injection, was established for 18 and 25 patients, respectively.

**Figure 5:**
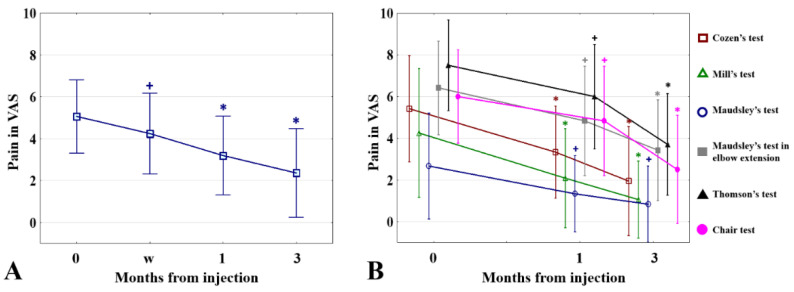
Change in pain intensity during follow-up measured in visual analog scale (VAS): (**A**) mean and SD pain intensity for a current day; (**B**) mean and SD pain intensity during provocation tests. w: one week; ^+^ *p* < 0.05 (compared to baseline); * *p* ≤ 0.001 (compared to baseline).

There was a significant decrease in pain intensity measured with the VAS at all follow-up points and both the mean values for the current day and during the provocation tests had improved. The decrease between the baseline pain and consecutive periods was: after one week 0.82 (SD = 2.10, *p* < 0.05), after one month 1.87 (SD = 2.3, *p* ≤ 0.001), and after three months 2.70 (SD = 2.73, *p* ≤ 0.001), see Figure 5. The values for each follow-up are presented in Supplementary Table S2.

**Figure 6:**
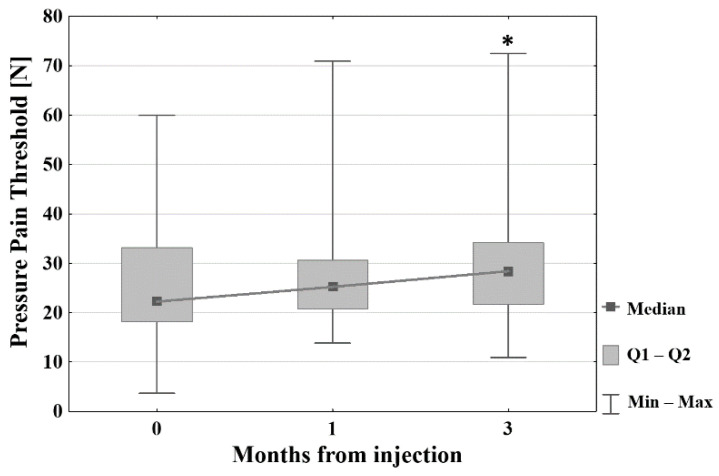
Result of pressure pain threshold assessment before, 1 and 3 months after intervention. * *p* < 0.05 (PPT 0 vs. PPT 3).

The pressure pain threshold assessment showed a decrease in local tenderness by increasing the force needed to induce pain by 2.68 (SD = 9.27, *p* = 0.102) and 5.06 (SD = 11.98, *p* < 0.05), after one and three months, respectively. The values are presented in Figure 6.

Three months after injection, most of the selected muscle groups improved in strength but a significant difference was only observed among the grip, elbow flexion, wrist extension, and forearm pronation strength (see Table 3).

**Figure 7:**
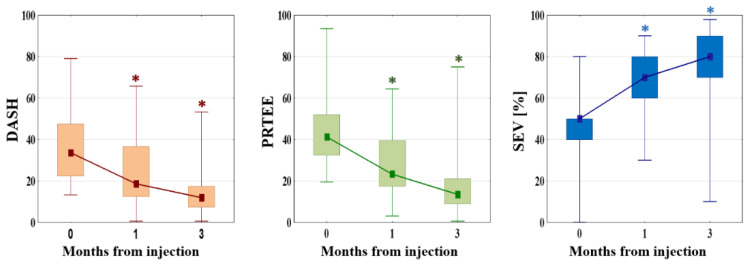
Results of DASH, PRTEE, and SEV scores before, 1, and 3 months after intervention. Square: median; box: Q1–Q2; whisker: MIN–MAX; * *p* ≤ 0.001 (comparison with baseline value); DASH: Disability of Arm, Shoulder and Hand questionnaire; PRTEE: Patient-rated tennis elbow evaluation; SEV: subjected elbow value.

Scores from both the functional questionnaires decreased significantly during the follow-up, which indicates a functional improvement. The DASH score decreased by 12.50 (SD = 10.71, *p* ≤ 0.001) and 22.11 points (SD = 16.52, *p* ≤ 0.001) after one and three months, respectively. The PRTEE score decreased by 17.25 (SD = 15.06, *p* ≤ 0.001) and 27.28 (SD = 21.28, *p* ≤ 0.001) points after one and three months, respectively. The SEV showed a significant increase from 47.50% (SD = 17.36) before treatment to 67.17% (SD = 14.84, *p* ≤ 0.001) after one month, and 73.77% (SD = 21.04, *p* ≤ 0.001) at the final follow-up. A comparison between the different time points is presented in Figure 7.

### 3.3. Correlation between Biologically Active Compounds in PRP and Treatment Efficacy

A significant moderate positive Pearson’s correlation was found between the PLT concentration in the PRP and a decrease in pain intensity at the 3rd month after injection (*r* = 0.56, *p* ≤ 0.001). There was also a positive low correlation between the PLT in the PRP and a PPT improvement (*r* = 0.46, *p* < 0.05). No correlation was found between the WBC or RBC content in the PRP and pain improvement at any point in the follow-up.

Between the growth factors and a pain decrease, only several significant correlations were found, but most of them were low. The concentration of EGF in the PRP had a positive moderate Pearson’s correlation with a VAS improvement three months after injection (*r* = 0.51, *p* < 0.05). Significant low positive correlations were found between pain decrease after three months and the VEGF (*r* = 0.36, *p* < 0.05), PDGF-AA (*r* = 0.37, *p* < 0.05) and PDGF-BB (*r* = 0.44, *p* < 0.05). Additionally, a PPT improvement significantly correlated at a low level with the PDGF-BB concentration in the PRP after three months (*r* = 0.38, *p* < 0.05).

**Figure 8:**
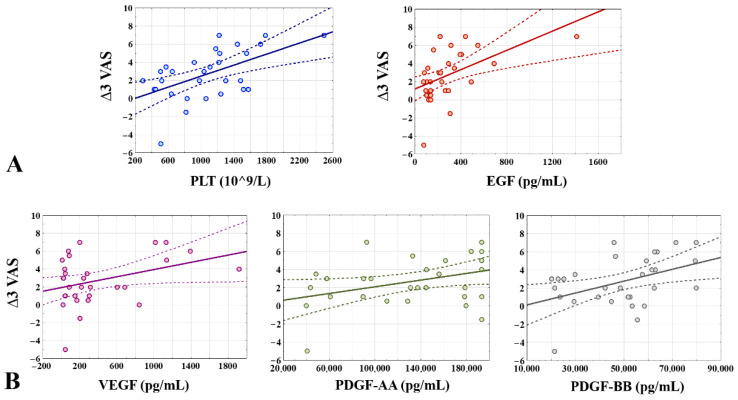
Significant positive Pearson’s correlations between biologically active compounds in PRP and size of reduction in pain intensity after 3 months from intervention (∆3 VAS = VAS 0 – VAS 3). (**A**) Significant moderate correlations (*r* = 0.56, *r* = 0.51, respectively). (**B**) Significant low correlations (*r* = 0.36, *r* = 0.37 and *r* = 0.44, respectively). PRP: platelet-rich plasma; PLT: platelets; EGF: epidermal growth factor; VEGF: vascular endothelial growth factor; PDGF-AA: platelet-derived growth factor-AA; PDGF-BB: platelet-derived growth factor-BB.

No significant correlation was found between the content of inflammatory cytokines and pain improvement. Selected significant positive correlations between the size of the decrease in pain intensity and biologically active compounds are presented in Figure 8.

Among the muscle groups whose strength improved significantly after three months from the injection (grip, elbow flexion, wrist extension, and forearm pronation), no correlation was found with the PLT, WBC, or RBC in the PRP. A significant positive low correlation was found between the EGF and a change in grip strength (*r* = 0.44, *p* <0.05), and also in wrist extension (*r* = 0.41, *p* < 0.05). The MCP-1 content in the PRP significantly negatively correlated with the improvement in strength during elbow flexion (*r* =−0.36, *p* < 0.05). 

A significant moderate positive correlation was found between the PLT content in the PRP and an SEV improvement at the 1st (*r* = 0.54, *p* < 0.05) and 3rd (*r* = 0.53, *p* < 0.05) month after injection. A significant positive low correlation was found between IL-18 in the PRP and a change in SEV after one month (*r* = 0.37, *p* < 0.05) but not after three months. No correlation was found between the cell content in the PRP and changes in the functional questionnaires score. A significant but low positive correlation was found between the EGF concentration in the PRP and a decrease in the PRTEE (functional improvement) after three months (*r* = 0.37, *p* < 0.05). Comparing the inflammatory cytokines content in the PRP and changes in the functional questionnaires, we found low negative correlations between IL-10 and a PRTEE decrease after one and three months (*r* =−0.37, *r* =−0.37, and *p* < 0.05, respectively), between IL-33 and a PRTEE decrease after one and three months (*r* =−0.38, *r* = 0.44, and *p* < 0.05, respectively), and between IL-17A and a PRTEE decrease after three months (*r* =−0.37, *p* < 0.05). A decrease in the DASH score after three months negatively correlated with the IL-33 concentration in the PRP (*r* =−0.39, *p* < 0.05).

### 3.4. Correlation between Treatment Efficacy and Baseline Characteristics

No correlations were found between a VAS improvement during the follow-up and age, BMI, pain sensitivity, or duration of symptoms. A significant moderate positive correlation was found between the baseline VAS and size of the VAS reduction after one and three months (*r* = 0.59, *p* < 0.05 and *r* = 0.63, *p* ≤ 0.001, respectively). A significant negative correlation was also found between the baseline SEV and size of the improvement in SEV after one and three months (*r* = −0.74, *p* < 0.05 and *r* =−0.59, *p* ≤ 0.001, respectively), a decrease in the VAS after three months (*r* =−0.54, *p* < 0.05), a decrease in the DASH after three months (*r* =−0.44, *p* < 0.05), or a decrease in the PRTEE after three months (*r* =−0.43, *p* < 0.05). Additionally, high baseline values of the functional questionnaires were significantly correlated with the size of reduction after three months (*r* = 0.73, *p* ≤ 0.001 for the DASH and *r* = 0.69, *p* ≤ 0.001 for the PRTEE). The above correlations suggest that the worse the symptoms are before treatment, the greater the improvement that could be expected.

## 4. Discussion

Due to the aim of the study, positive correlations were found between the size of pain reduction after three months from the intervention, measured by a VAS and several growth factor concentrations in PRP: EGF, VEGF, PDGF-AA and PDGF-BB. A significant positive correlation was also found between the size of the pain reduction and the concentration of PLT in the PRP. After three months, the EGF concentration in the PRP significantly correlated with an improvement in grip strength, wrist extension strength, and functional improvement size measured by the PRTEE. Some negative correlations were found between several inflammatory cytokines (IL-10, IL-33, and IL-17A) and changes in the functional questionnaires in the 3rd month after the intervention.

In our study, most patients were treated successfully after three months. Twenty patients (67%) reached a minimal clinically important difference in pain measured by the VAS, and in functional improvement measured by DASH. Even more patients (83%) reached a MCID in functional improvement measured by the PRTEE. Additionally, we established that the worse the pain, SEV or functional score had been at the beginning, the better the improvement that was obtained after three months.

Problems in assessing the effectiveness of PRP treatments start with the definition. Some researchers declare that PRP should be plasma with a platelet density above 1 million per µL [29]. In their review, Oudelaar et al., found that four among seven investigated commercially available systems were able to meet this criterion [19]. Although the mean PLT concentration in the PRP in our study was more than 1 million per µL (1,073,630 platelets per µL), only 17 out of the 30 samples met this criterion (range 289,000–2,491,000 platelets per µL). On the other hand, there are also researchers who believe that a platelet level > 200,000 per µL is sufficient to obtain a clinical effect [30]. All our PRP samples reached the above value. Additionally, significant differences in the content of WBC could lead to a product with completely different biological properties [19,31,32]. This differentiation made it necessary to propose a precise classification that would allow a reliable assessment of the PRP used. One of the first widespread classifications divided the PRP according to the leukocyte and fibrin content [33]. As this does not solve all the problems, subsequent classifications have proposed a division according to the leukocyte content, high (≥5 times the baseline) or low platelet concentration, and the presence of additional PRP activation [34]. Further classifications added new parameters that seemed to be significant, such as the description of the centrifugation process, the separation technique, the injection technique, the presence of light activation, and the presence of RBC in the final product [35]. A consensus has even been developed on what information should be described in a study using PRP [36]. The separation system used by us is one of the most frequently used, as it enables to obtain PRP with a high platelet density (4–6 folds than the baseline) in a reproducible manner [20]. According to the MARSPIL classification, in our study we used leukocyte-rich, red blood cells-rich, non-activated, 4–6 times greater than the baseline platelet-rich plasma, handmade with a single-spin centrifugation and injected with ultrasound guidance; what should be reported as M_(H)_, A_(A−)_, R_(RBC-R)_, S_(Sp1)_, P_(PL [4–6])_, I_(G+)_, L_(Lc-R[4–6]),_ L_(A−)_ [35]. The individual capital letters of the abbreviation in the above classification refers to: the preparation method (M), presence of activation (A), presence of red blood cells (R), number of spins (S), platelet concentration comparing to the baseline (P), presence of imaging guidance during injection (I), presence and concentration of leukocytes comparing to the baseline (L), and the presence of light activation (L), respectively. Each of the above should be clarified by adding the individual abbreviations below: whether it was prepared by an automated manner (machine: M) or handmade (H); the spin number (Sp1 or Sp2); red blood cells (RBCs; rich: RBC-R, poor: RBC-P); platelet concentration (PL: 2–3; PL: 4–6; PL: 6–8 and PL: 8–10 folds the baseline); leukocyte rich (Lc-R) or poor (Lc-P) and the range; activated (A+) or not (A−); light activated (L+) or not (L−), and injected with imaging guidance (G+) or not (G−) [35]. The above classification shows well how heterogeneous this treatment method is. According to the latest classification from 2020 proposed by Kon et al., and accepted by many researchers by consensus, the PRP used in our study should be reported as: 210-14-00. The digits refer to the whole blood PLT concentration (where “2” means 200,000–300,000 platelets/µL), PLT concentration in PRP (where “10” means 1,000,000–1,100,000 platelets/µL), presence of RBC in PRP (where “1” means > 1 × 10^6^/μL), increase in WBC from baseline (where “4” means 4.1–5.0 × baseline), presence of activation (where “0” means no external activation), and the addition of calcium (where “0” means no addition), respectively [37].

Several investigations were conducted to evaluate the content of the biologically active compounds in PRP obtained by various methods [19]. Significant positive correlations were found between PLT concentrations in PRP and EGF, VEGF, PDGF, TGF-β1 [20,38,39] and these and other growth factors and cytokines are involved in the proper healing of soft tissues. They interact through stimulation of the migration of cells such as neutrophils, monocytes, and fibroblasts to the site of injury (PDGF), stimulation of extracellular matrix production (PDGF), stimulation of cell migration, proliferation, and differentiation (FGF, EGF, HGF, TGF-β1), as well as stimulation of angiogenesis (VEGF, HGF) [12,40,41]. In our study, a positive relationship between the above growth factors and clinical improvement was found with the EGF, VEGF, and PDGF, which could support their positive effect on the healing processes in vivo. Despite expectations, no relationship was found between any clinical outcome and TGF-β1. Inflammatory cytokines also could positively influence tissue healing by the attraction of macrophages and neutrophils (IL-8, MCP-1), the stimulation of reepithelialization (IL-8), inhibition of inflammation and scar formation (IL-10), stimulation of keratinocyte or fibroblast proliferation, and the regulation of immune responses (IL-1, IL-6, TNF-α) [12]. An increased amount of various inflammatory cytokines could also have a negative impact on tissue healing leading to an excessive inflammatory reaction. In our study, we found only one positive correlation between the inflammatory cytokine (IL-18) and clinical effect (increase in SEV) after one month, but it was no longer present after three months; however, some negative correlations were found between the clinical outcomes and IL10, IL-33 and IL-17A. Unfortunately, the numerous interactions between growth factors and cytokines in varying concentrations and proportions lead to almost unpredictable clinical effects inside the human body. Recent studies have demonstrated a significant role of IL-17 in the pathophysiology of tendinopathies. IL-17 has been shown to mediate the inflammatory response by stimulating the production of pro-inflammatory cytokines and tissue remodeling in human tenocytes. It is proposed as a potential therapeutic target in tendinopathy [42,43]. In our study, we found a negative correlation between IL-17A in the PRP and a functional improvement examined with the PRTEE questionnaire. This result may support the thesis that this cytokine plays an important role in lateral elbow tendinopathy.

Many in vitro, animal and human studies have been conducted to evaluate the effects of PRP on soft tissues and it has been effectively used for wound healing disorders, bone union disorders, sports injuries, osteoarthritis, rheumatoid arthritis and chronic overuse injuries such as different tendinopathies [44,45,46].

Niemiec et al., in their meta-analysis of 26 studies with PRP injection for lateral epicondylopathy, established the treatment as effective in the achievement of MCID during all points of follow-up (4–104 weeks) [16]. In another meta-analysis based on 16 studies, Chen et al., found that despite a significant clinical improvement, they were unable to recommend for or against the use of PRP for lateral epicondylitis. This was due to the small number of comparable studies, a lack of quantification of the specific PRP content, considerable heterogeneity between the randomized control trials, and most effect sizes being equivocal within the framework of two estimated MCID values [47]. Another point of view was presented by de Vos et al., in their systematic review based on six studies, in which the authors stated that there was strong evidence that PRP is not effective in treating chronic lateral elbow tendinosis [17]. Based on our experience and on the available literature, we believe that injection of PRP brings relief to a large number of patients with lateral elbow tendinopathy. Our findings lead to the assumption that a better result is expected when using a PRP with a higher platelet concentration. The use of preparations with a low content of platelets can be confusing and could be the reason for inconclusive results of systemic reviews and meta-analyses. On the other hand, research increasingly shows that there is no significant difference in the clinical effect between the different types of PRP [15]; therefore, it cannot be ruled out with certainty that the injection technique itself is of great importance in achieving clinical improvement [48]. Due to the lack of comparison with the control group, it cannot be excluded for sure, that the positive results of the PRP treatment in our study were related to the injection technique itself, a placebo-effect or to the recommended post-treatment rehabilitation protocol.

Only a few studies have been conducted to assess the correlation of growth factors in PRP with the clinical effect of the treatment. No previous study has assessed the correlation of inflammatory cytokines with clinical effect and to our knowledge, only one study has evaluated the correlation of the clinical effect of patients with tennis elbow treated with PRP injection and the growth factors within it. Lim et al., in their randomized controlled trial, evaluated 156 patients with lateral epicondylitis divided into two groups [49]. One group received a single injection of 2 ml of PRP and the control group was treated by physical therapy. For the final analysis, they evaluated 55 patients after receiving PRP injections and 50 in the control group. PDGF-AB, PDGF-BB, TGF-β, EGF, EGF, and IL-1b were among the growth factors assessed in PRP samples. During the follow-up at three and six months, pain intensity was evaluated with a VAS, and functional results with the Modified Mayo Clinic Performance Index. Additionally, a magnetic resonance imaging (MRI) was performed before treatment and after six months, to evaluate significant changes. At the final follow-up visit, VAS, MAYO, and MRI improvements were observed in both groups; however, it was significant only in the PRP group. No complications or adverse events were observed. The authors found a significant low correlation between the WBC and VAS improvement (*r* = 0.318) and an even lower correlation between the TGF-β and MAYO improvement (*r* = 0.275). Additionally, some positive significant correlations between an MRI improvement and both VEGF and TGF-β were reported [49]. As in our study, positive treatment results were obtained and positive correlations between the clinical outcomes and the content of the biologically active compounds in PRP were found; however, the authors showed a correlation between the treatment efficacy and TGF-β, which our study did not confirm. There were some small differences between the PRP used in our and their study. Similarly to our study, the authors used leukocyte-rich platelet-rich plasma, but they obtained a final mean platelet concentration approximately 6.87 times higher than the baseline compared to our 4.38. They also used 10% calcium chloride for the platelet activation just before injection. This kind of activation is responsible for a more rapid release of growth factors from the platelet α-granules. We decided to use natural platelet activation in vivo, by the contact with the native collagen present in the connective tissue for a more stable cytokine release [50]. Similar to our study, they also used approximately 2 ml of 1% lidocaine and administered a single injection into the tendon attachment. In their study, the VAS improvement at the final follow-up was higher than in our study (with a decrease in pain about 4.06 points in VAS recalculated to a 0–10 scale, compared to our 2.7 points). This could have been influenced by the longer observation period but the drop-outs in follow-up were not clearly stated in the publication. The functional improvement measured by the MAYO score was similar to those measured by the DASH and PRTEE in our study.

Another study that presented a relationship between the growth factors in PRP and the clinical outcome of its injection is the study published by Kim et al. [51]. The authors randomly divided 30 patients with rotator cuff tendinopathy into a group receiving a 2 mL injection of PRP or to a group undertaking only strengthening exercises. Pain and functional improvement were assessed with the American Shoulder and Elbow Surgeons (ASES), Constant–Murley score, and numeric rating scale (NRS) during 6, 12, and 24 weeks of follow-up. The PRP samples were analyzed to establish the concentrations of TGF-β1, TNF-α, PDGF-AA, PDGF-AB, PDGF-BB, VEGF, EGF, IGFBP-1, IL-1β, and IL-8. The authors found a significant correlation between cytokines and clinical outcomes only at 12 weeks of follow-up between the IL-1β and a change in the Constant–Murley score (*p* = 0.046), and between the TGF-β1 and a change in the NRS at 12 weeks (*p* = 0.048). The cut-off values to predict a meaningful improvement were established at 5.19 pg/mL in the IL-1β and 61.79 μg/mL in the TGF-β1. In their study, the authors concluded that better clinical outcomes for rotator cuff tendinopathy were found in patients who received PRP with IL-8 and TGF-β1 above these cut-off values than for the exercise group. The biggest limitation of this study was a relatively small group with a high percentage of drop-outs during the follow-up stages [51]. Again, our findings do not support a significant relationship between IL-8 or TGF-β1 and clinical outcomes. This may, however, be due to significant methodological differences between the two studies.

Louis et al., showed results of a randomized blinded controlled trial comparing PRP and hyaluronic acid injection into osteoarthritic knees [52]. Fifty four patients were divided into comparable groups and were assessed by the Western Ontario and McMaster Universities Arthritis Index (WOMAC) score at a baseline and at one, three, and six months. The VEGF, PDGF-AB, and TGF-β1 contents of injected PRP were assessed. The authors found clinical improvement in both groups but also a correlation between a worsening in the WOMAC score and high concentrations in PRP of both TGF-β1 and PDGF-AB [52]. In our study, we found a positive correlation between the VEGF or PDGF concentrations in PRP and clinical outcomes; however, the results of osteoarthritis treatment cannot be directly translated into a treatment for tendinopathy due to significant differences in the pathophysiology of both diseases.

Rodrigues et al., in their randomized controlled trial, evaluated the relationship between growth factors in PRP and hair growth parameters on patients with alopecia treated with four subcutaneous PRP injections or a placebo [53]. PDGF, EGF and VEGF were measured in PRP samples. The authors demonstrated a significant increase in hair count, hair density and percentage of anagen hairs in the PRP group versus in the control group, without any correlation with platelet or growth factor concentrations in the PRP [53].

In our study, we also found a positive correlation between symptoms’ severity at the beginning, and the size of improvement in both pain intensity and functional outcomes. Haahr et al., tried to assess the prognostic factors of 266 lateral epicondylitis cases treated with minimal occupational intervention during a one-year follow-up. They found that a poor prognosis was related to manual work and a high baseline pain [4]. In contrast, in our study, the greater the pain complaints were at the baseline, the greater the improvement that was obtained. This may lead to the assumption that patients with low severity will benefit more from a rehabilitation program, and those with symptoms of significant severity will benefit more from the earlier implementation of injection therapy.

The strengths of our study include a wide spectrum of clinical evaluation of the PRP injection treatment efficacy in patients with lateral elbow enthesopathy. That includes an assessment of the mean pain for the current day, pain intensity during provocation tests, tenderness of the affected area, the strength of various muscle groups and a functional assessment using questionnaires. The wide range of growth factors and inflammatory cytokines analyzed far exceeds the amount presented in other studies. The relationship of most of the studied molecules in PRP and their clinical effect was investigated for the first time.

The limitations of the study include the relatively small group of patients, the lack of MRI control before and after treatment, and not considering some of the existing cytokines and growth factors that may interfere with the healing process. The study shows the correlation of clinical outcomes and individual growth factors or inflammatory cytokines, but does not take into account the possibility of their mutual influence, both stimulating and inhibiting. Unfortunately, much larger groups of subjects would be needed to reliably estimate the clinical efficacy of the various combinations of cytokines. Another limitation of the presented study is the lack of results from the control groups. These will be provided after the long-term end of further data collection. Additionally, the study has a relatively short observation time; however, according to our experience and the experience of other researchers, waiting for any clinical pain improvement for more than three months is often unacceptable for patients who start seeking help elsewhere and often drop out of the study [18].

## 5. Conclusions

The injection of autologous platelet-rich plasma is a safe and effective treatment method for patients with lateral elbow tendinopathy. Our findings showed that PRP with a higher platelet concentration is correlated with a better decrease in pain during three months of follow-up. For the first time in the literature, we showed a significant positive correlation of EGF, VEGF and PDGF concentrations in PRP and the size of pain reduction among those patients. After three months, the EGF concentration in the PRP significantly correlated with an improvement in grip strength, strength of wrist extension and size of functional improvement measured by a PRTEE. Negative correlations were found between the IL-10, IL-33 and IL-17A concentrations in the PRP and the change in the functional questionnaires in the third month after intervention. Further studies are needed in a larger group, with a longer follow-up, other tendinopathies, and with the use of different types of PRP to reliably assess the relationship between the clinical effect and the content of the biologically active compounds in injected PRP.

## Figures and Tables

**Table 1:** The main baseline characteristics and the values of the pretreatment clinical evaluation.

Patients’ Characteristics		Baseline Evaluation Values	
Age *	49.0 (6.12)	VAS 0–10 *	5.07 (1.76)
Sex (*n* (%))		*Cozen’s test*	5.42 (2.55)
Female	15 (50)	*Mill’s test*	4.25 (3.09)
Male	15 (50)	*Maudsley’s test*	2.67 (2.54)
BMI *	27.5 (4.67)	*Maudsley’s test with extended elbow*	6.42 (2.24)
Hand dominance (*n* (%))		*Thomson’s test*	7.50 (2.18)
Right	27 (90)	*Chair test*	6.00 (2.24)
Left	3 (10)	PPT in N *	25.38 (11.76)
Affected elbow (*n* (%))		Pain sensitivity in N *	42.99 (16.99)
Right	19 (63)	Strength of muscle groups *	
Left	11 (37)	*Grip* in MAX kg	32.45 (14.28)
Dominant hand affected (*n* (%))	22 (73)	*Elbow flexion in N*	234.97 (90.34)
Physical labor (*n* (%))	15 (50)	*Elbow extension in N*	175.96 (66.71)
Regular sports activity (*n* (%))	14 (47)	*Wrist flexion in N*	159.03 (61.28)
Current smokers (*n* (%))	3 (10)	*Wrist extension in N*	116.44 (52.47)
Alcohol consumption (*n* (%))		*Forearm supination in N*	26.05 (12.13)
occasionally (≤1 dose per week)	22 (73)	*Forearm pronation in N*	36.64 (18.35)
not at all	8 (27)		
Duration of symptoms in months		DASH *	37.28 (17.17)
(mean (SD))	17.33 (25.38)	PRTEE *	45.98 (19.17)
(median (MIN–MAX))	5 (3–120)	SEV *	47.50 (17.36)

* The values expressed as arithmetic mean (standard deviation). BMI: body mass index; VAS: visual analogue scale; PPT: pressure pain threshold; DASH: Disability of Arm, Shoulder and Hand questionnaire; PRTEE: Patient-Rated Tennis Elbow Evaluation; SEV: subjected elbow value.

**Table 2:** Differences in cellular content between whole blood and PRP. Values are presented as arithmetic mean (standard deviation).

	Whole Blood	PRP	*p*
PLT [10^3^/μL]	252.72 (60.34)	1073.63 (498.63)	≤0.001
WBC [10^3^/μL]	6.57 (1.36)	30.01 (9.74)	≤0.001
*Neutrophiles*	4.05 (1.23)	12.70 (6.77)	≤0.001
*Lymphocytes*	1.95 (0.52)	14.12 (5.12)	≤0.001
*Monocytes*	0.39 (0.1)	2.80 (1.09)	≤0.001
*Eosinophiles*	0.15 (0.12)	0.17 (0.17)	0.64
*Basophiles*	0.03 (0.02)	0.20 (0.12)	≤0.001
RBC [10^6^/μL]	4.89 (0.41)	1.03 (0.77)	≤0.001

PRP: platelet-rich plasma; PLT: platelets; WBC: white blood cells; RBC: red blood cells. The significance of the Student’s *t*-test for paired samples comparison is shown as the *p*-value.

**Table 3:** The strength of individual muscle groups before treatment and during follow-up visits.

	Baseline	after 1 Month	*p* ^1^	after 3 Months	*p* ^2^
Grip (MAX kg)	32.45 (14.28)	33.43 (13.63)	0.73	35.96 (14.46)	<0.05
Elbow flexion (N)	234.97 (90.34)	236.83 (91.14)	0.81	246.44 (84.57)	<0.05
Elbow extension (N)	175.96 (66.71)	172.07 (54.51)	0.43	172.17 (56.83)	0.39
Wrist flexion (N)	159.03 (61.28)	151.81 (48.27)	0.30	162.07 (59.05)	0.40
Wrist extension (N)	116.44 (52.47)	121.50 (51.14)	0.80	140.46 (49.45)	<0.05
Forearm supination (N)	26.05 (12.13)	24.71 (10.54)	0.31	29.05 (15.74)	0.07
Forearm pronation (N)	36.64 (18.35)	40.24 (17.93)	0.09	46.44 (20.15)	≤0.001

Values are presented as arithmetic mean (standard deviation). *p*-value represents significance of comparison between time periods: *p*^1^ = baseline vs. 1st month; *p*^2^ = baseline vs. 3rd month.

## Data Availability

The data used to support the findings of this study are available in the Supplementary Files. All data that may violate the ethics or privacy of subjects are available from the corresponding author upon reasonable request.

## Supplementary Materials

The following supporting information can be downloaded at: https://www.mdpi.com/article/10.3390/jcm11133687/s1, Table S1: Growth factors and inflammatory cytokines concentrations in all PRP samples [pg/mL]; Table S2: Outcome measurements before (0) and during follow-up (1, and 3 months).
