# Supplementary material for: Effectiveness of Lateral Elbow Tendinopathy Treatment Depends on the Content of Biologically Active Compounds in Autologous Platelet-Rich Plasma
Source: J Clin Med. 2022 Jun 27;11(13):3687. doi: 10.3390/jcm11133687 (PMC9267331; doi:10.3390/jcm11133687)
Supplement: Supplementary file 1 [file jcm-11-03687-s001.zip › jcm-1761412-supplementary.pdf]

Supplementary Table S1. Growth factors and inflammatory cytokines concentrations in all PRP samples [pg/ml].

	**Mean**	**SD**	**Median**	**Q1 – Q3**
Transforming Growth Factor-β1, free active	382.46	256.01	337.36	257.65 – 443.32
Epidermal growth factor	282.77	263.27	219.13	116.43 – 340.91
Fibroblast Growth Factor- basic	2826.10	8119.49	315.98	254.70 – 450.50
Vascular endothelial growth factor	384.69	487.88	197.10	40.95 – 602.85
Hepatocyte growth factor	497.65	1027.87	208.98	156.05 – 290.69
Platelet-Derived Growth Factor-AA	132665.17	54524.17	140920.85	90062.55 – 183956.91
Platelet-Derived Growth Factor-BB	49160.61	18377.38	52190.81	29826.86 – 62573.63
Interleukin-1β	76.16	70.67	46.69	30.61 – 100.44
Interferon-α2	39.76	28.85	27.13	16.16 – 50.14
Interferon-γ	7.10	3.73	4.87	4.87 – 7.45
Tumor Necrosis Factor α	33.76	27.29	23.81	13.02 – 42.72
Monocyte Chemoattractant Protein-1	128.18	125.01	94.22	63.97 – 151.18
Interleukin-6	20.23	12.05	15.24	12.16 – 22.34
Interleukin-8	126.66	133.63	61.38	46.77 – 157.73
Interleukin-10	20.10	9.78	17.28	12.88 – 24.98
Interleukin-12p70	21.09	11.33	16.78	11.47 – 27.80
Interleukin-17A	3.22	1.70	2.58	1.94 – 4.28
Interleukin-18	411.35	293.41	334.12	185.68 – 507.57
Interleukin-23	77.14	24.03	65.97	65.97 – 65.97
Interleukin-33	177.51	100.79	161.02	95.95 – 229.86

PRP: Platelet-rich plasma; SD: standard deviation; Q1 – Q3: the first and the third quartile.

Supplementary Table S2. Outcome measurements before (0) and during follow-up (1, 3 month).

	**Mean**	**SD**	**Median**	**Q1 – Q3**	***p* (vs. 0)**
0 VAS	5.07	1.76	5.00	4 – 6	
1 VAS	3.2	1.88	3.00	2 – 4	≤ 0.001
3 VAS	2.37	2.12	2.00	1 – 3	≤ 0.001
0 Cozen's test	5.42	2.55	5.00	5 – 7.5	
1 Cozen's test	3.33	2.21	2.5	2.5 – 5.0	≤ 0.001
3 Cozen's test	1.95	2.58	0.5	0 – 2.5	≤ 0.001
0 Mill's test	4.25	3.09	5.00	2.5 – 7.5	
1 Mill's test	2.08	2.37	2.5	0 – 2.5	≤ 0.001
3 Mill's test	1.2	1.99	0	0 – 2.5	≤ 0.001
0 Maudsley's test	2.67	2.54	2.50	0 – 5.0	
1 Maudsley's test	1.33	1.83	0	0 – 2.5	< 0.05
3 Maudsley's test	0.95	1.90	0	0 – 1.0	< 0.05
0 Maudsley's test in extended elbow	6.42	2.24	7.50	5 – 7.5	
1 Maudsley's test in extended elbow	4.83	2.62	5.0	2.5 – 7.5	< 0.05
3 Maudsley's test in extended elbow	3.4	2.45	2.5	1.5 – 5.0	≤ 0.001
0 Thomson's test	7.5	2.18	7.5	7.5 – 10	
1 Thomson's test	6	2.50	7.5	5 – 7.5	< 0.05
3 Thomson's test	3.73	2.5	4.00	2.5 – 5.0	≤ 0.001
0 Chair test	6	2.24	6.25	5 – 7.5	
1 Chair test	4.83	2.62	5.00	2.5 – 7.5	< 0.05
3 Chair test	2.65	2.63	2.5	0 – 5.0	≤ 0.001
0 PPT [N]	25.38	11.76	22.21	18.14 – 33.15	
1 PPT [N]	28.06	11.81	25.20	20.69 – 30.60	0.102
3 PPT [N]	30.44	14.28	28.39	21.67 – 34.13	< 0.05
0 SEV	47.50	17.36	50	40 – 50	
1 SEV	67.17	14.84	70	60 – 80	≤ 0.001
3 SEV	73.77	21.04	80	70 – 90	≤ 0.001
0 DASH	37.28	17.17	33.75	22.5 – 47.50	
1 DASH	24.78	16.04	18.75	12.5 – 36.67	
3 DASH	15.17	12.32	12.08	7.5 – 17.50	≤ 0.001
0 PRTEE	45.98	19.77	41.25	32.5 – 52.00	
1 PRTEE	28.73	17.37	23.25	17.5 – 39.50	≤ 0.001
3 PRTEE	18.71	16.30	13.5	9 – 21.00	≤ 0.001
0 grip strength	32.5	14.28	30.0	22.00 – 44.0	
1 grip strength	33.4	13.63	30.0	22.00 – 46.0	0.728
3 grip strength	35.8	15.13	36.0	22.00 – 50.0	< 0.05
0 wrist extension strength	116,4	52,47	109,3	78,45 – 158.9	
1 wrist extension strength	121,5	51,14	107,9	90,22 – 151.0	0.804
3 wrist extension strength	140,6	50,01	148,1	101,01 – 177.5	< 0.05

0 wrist flexion strength	159,0	61,28	146,6	118,66 – 199.1	
1 wrist flexion strength	151,8	48,27	141,7	116,70 – 193.2	0.304
3 wrist flexion strength	162,7	58,37	154,9	114,74 – 200.1	0.501
0 forearm supination strength	26,1	12,13	23,5	16,67 – 32.4	
1 forearm supination strength	24,7	10,54	23,5	17,65 – 33.3	0.311
3 forearm supination strength	29,8	16,19	23,5	18,63 – 36.3	0.221
0 forearm pronation strength	36,6	18,35	27,9	24,52 – 50.0	
1 forearm pronation strength	40,2	17,93	33,3	26,48 – 55.9	0.094
3 forearm pronation strength	47,6	20,46	42,2	28,44 – 62.8	≤ 0.001
0 elbow extension strength	176,0	66,71	160,3	128,47 – 215.7	
1 elbow extension strength	172,1	54,51	155,4	129,45 – 207.9	0.433
3 elbow extension strength	170,2	58,46	155,9	125,53 – 217.7	0.600
0 elbow flexion strength	235,0	90,34	220,2	147,10 – 302.0	
1 elbow flexion strength	236,8	91,14	208,4	165,73 – 313.8	0.805
3 elbow flexion strength	238,7	79,80	215,7	170,64 – 317.7	< 0.05

PPT: Pressure Pain Threshold; SD: standard deviation; Q1 – Q3: the first and the third quartile; SEV: Subjected Elbow Value; DASH: Disability of Arm, Shoulder and Hand Questionnaire; PRTEE: Patient-rated tennis elbow evaluation.
